# Systemic racism and mental health services: the time is now

**DOI:** 10.1192/bjb.2020.108

**Published:** 2020-12

**Authors:** Roxanne Keynejad, Alice Debelle, Julia Ogunmuyiwa, Arleen Elson, Marilia Calcia

**Affiliations:** Clinical Research Training Fellow and ST4 in General Adult Psychiatry, King's College London. email: roxanne.1.keynejad@kcl.ac.uk; ST5 Higher Trainee in Child & Adolescent Psychiatry, South London and Maudsley NHS Foundation Trust; ST5 Higher Trainee in General Adult Psychiatry, South London and Maudsley NHS Foundation Trust; Diversity and Inclusion Human Resources Lead, South London and Maudsley NHS Foundation Trust; Consultant Liaison Psychiatrist, South London and Maudsley NHS Foundation Trust

The killing of George Floyd refocused global attention on racial injustice. UK Covid-19 death rates are highest among Black, Asian and minority ethnic (BAME) groups,^1^ with systemic racial inequality a central cause.^2^ Although BAME people face many inequalities, Black people's unique experiences require particular attention.^3^

In mental healthcare, we must consider links between psychiatric symptoms and experiencing racism, systemic racism within services and medico-legal interfaces. The *Delivering Race Equality in Mental Health Care* (DRE) agenda^4^ was prompted by the 1998 death of David Bennett, an African–Caribbean patient, following in-patient restraint. DRE prioritised reducing fear of services, developing culturally appropriate therapies, and engaging BAME groups and patients in training, policy development, service planning and provision.

A DRE progress review^5^ advocated dedicated community development workers, engagement projects, training, clinical trailblazers and measuring progress. Patients, professionals, campaigners and academics attributed continued race inequality in mental healthcare post-DRE to institutional racism, inadequate training, poor system design and lack of an empowering culture.^6^

In our experience, DRE is not prioritised. Core training competencies of ‘cultural diversity’, evaluating institutional prejudices, respect for diversity and evaluating biases are neglected. Despite a strong position statement,^7^ racism is absent from MRCPsych examinations. Static e-learning modules replace interactive, in-person training that could stimulate engagement, discussion and reflection. Black leadership and collaboration with Black community stakeholders are lacking in mental health trusts serving largely Black populations.

Mental health service leaders must role-model, reflecting on personal and workplace unconscious biases.^8^ We should each ask of our own organisation, how equitable is provision? Do Black patients have equal access to psychological therapies and specialist services? Are their perspectives represented? Do we foster dialogue with local communities? Do Black staff experience disproportionate bullying and harassment? Can they speak up if safeguards are lacking? Identifying and ameliorating clinical inequalities should drive research, so that treatments meet Black patients’ needs.^9^

‘We do not need another review, or report, or commission to tell us what to do’ about race inequality in the UK,^10^ nor in mental healthcare. To address mistrust, services must acknowledge and address inequalities experienced by Black patients. We welcome RCPsych's appointment of presidential race equality leads and hope they will forge multidisciplinary alliances to mainstream anti-racism across mental health professionals.

It should not have taken a death to trigger the biggest race equality focus in mental health services’ history, nor should it have been so rapidly forgotten. Black stakeholders must be empowered to occupy positions of influence, but it is not Black staff or patients’ responsibility to effect change; organisations must be accountable. In a mostly White-led profession, tackling systemic racism will inevitably cause discomfort. Mental healthcare, with its recognition of transference and countertransference, and prioritisation of supervision, reflection and psychotherapeutic skills, is well-placed to lead the difficult discussions the health service needs. Silence is not neutral. The time is now.
